# Supplementary material for: Recurrence Patterns after Surgery in Patients with Different Endometriosis Subtypes: A Long-Term Hospital-Based Cohort Study
Source: J Clin Med. 2020 Feb 11;9(2):496. doi: 10.3390/jcm9020496 (PMC7073694; doi:10.3390/jcm9020496)
Supplement: Supplementary file 1 [file jcm-09-00496-s001.pdf]

Journal of
Clinical Medicine

MDPI

Article

Recurrence patterns after surgery in patients with different endometriosis subtypes: a long-term hospital-based cohort study

Supplemental tables and figures

Figure S1. Time to first recurrence for all patients (*n* = 322)

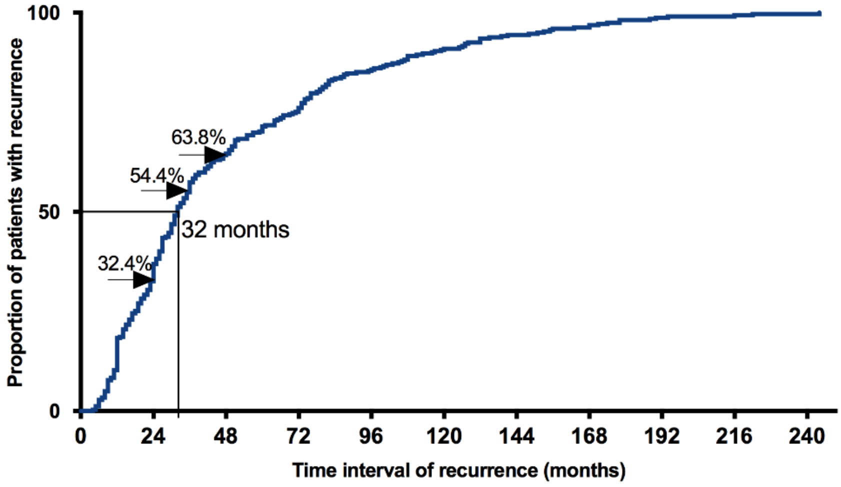

Legend Fig S1: The median time to recurrence is 32 months. The black arrows show the percentage of patients with recurrence at 24 months, 36 and 48 months, respectively.

Figure S2. Time to first, second, third and fourth recurrence

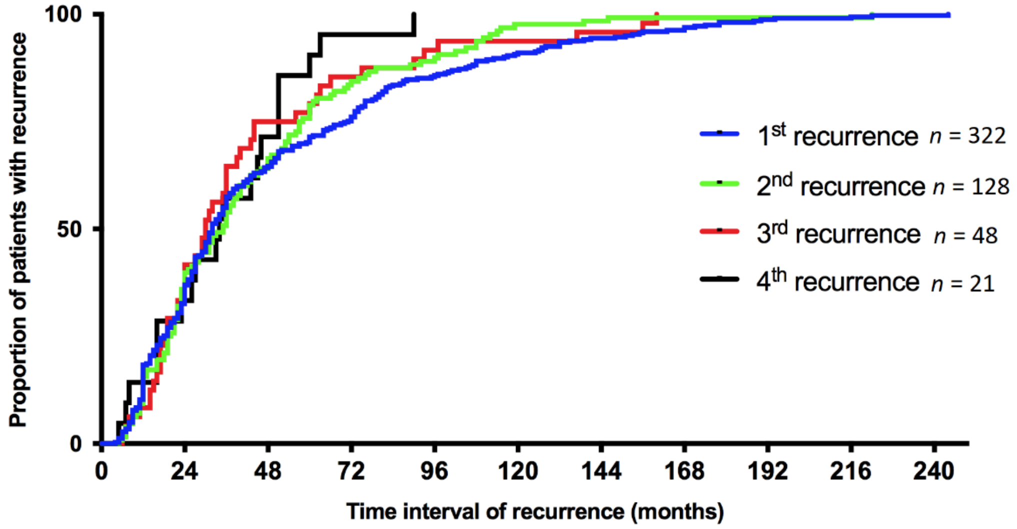

Legend Fig. S2: The time from the first to second (1st recurrence), second to third (2nd recurrence), third to fourth (3rd recurrence) and fourth to fifth (4th recurrence) surgery is illustrated in different colors. The median time to the first, second, third and fourth recurrent surgery is 32, 35, 30 and 34 months, respectively. No statistically significant difference was observed.

Table S1. Evolution of endometriosis from second to third surgery.

Second surgery / Third surgery	SUP (*n* = 11); median time to recurrence (min-max)	OMA (*n* = 48); median time to recurrence (min-max)	DIE (*n* = 32); median time to recurrence (min-max)
SUP	6 (54.5%); 40.5 (9-125)	3 (10.7%); 112 (23-119)	7 (21.9%); 27 (7-61)
OMA	0 (0%)	25 (52.1%); 35 (8-222)	9 (28.1%); 29 (12-89)
DIE	5 (45.5%); 23 (13-70)	17 (35.4%); 43 (6-114)	15 (46.9%); 54 (12-146)
Unknown subtype	0	3 (6.2%); 21 (5-38)	1 (3.1%); 23

Note: In each cell, the number of cases evolving into SUP, OMA, DIE or unknown lesion subtype at recurrent surgery and their percentage is given. Abbreviations: SUP, superficial peritoneal endometriosis; OMA, ovarian endometrioma; DIE, deep infiltrating endometriosis; Min-Max, Minimum-Maximum

Table S2. Evolution of endometriosis from third to fourth surgery.

Third surgery / Fourth surgery	SUP (*n* = 8); median time to recurrence (min-max)	OMA (*n* = 15); median time to recurrence (min-max)	DIE (*n* = 13); median time to recurrence (min-max)
SUP	4 (50%); 29 (11-90)	1 (6.7%); 137	2 (15.4%); 46 (30-62)
OMA	1 (12.5%); 17	9 (60.0%); 17 (6-156)	5 (38.5%); 39 (27-93)
DIE	3 (37.5%); 29 (24-160)	5 (33.3%); 32 (15-63)	6 (46.2%); 40 (24-141)
Unknown	0	0	0

Note: In each cell, the number of cases evolving into SUP, OMA, DIE or unknown lesion subtype at recurrent surgery and their percentage is given. Abbreviations: SUP, superficial peritoneal endometriosis; OMA, ovarian endometrioma; DIE, deep infiltrating endometriosis; Min-Max, Minimum-Maximum

© 2020 by the authors. Submitted for possible open access publication under the terms and conditions of the Creative Commons Attribution (CC BY) license (http://creativecommons.org/licenses/by/4.0/).
